# IRF1 in Adipocytes Is Associated with Insulin Signaling and Mitochondrial Homeostasis in Diet-Induced Obesity

**DOI:** 10.3390/ijms27167332

**Published:** 2026-08-17

**Authors:** Airan Zhu, Ying Zhou, Kaili Ren, ChongXiu Sun

**Affiliations:** Collaborative Innovation Center for Cardiovascular Disease Translational Medicine, Nanjing Medical University, 101 Longmian Avenue, Jiangning District, Nanjing 211166, China; airan.zhu@stu.njmu.edu.cn (A.Z.); yingzhou5002@163.com (Y.Z.); m15535830045@163.com (K.R.)

**Keywords:** IRF1, adipocyte, insulin signaling, mitochondrial homeostasis, obesity, energy expenditure, glucose metabolism, lipid metabolism

## Abstract

Obesity-associated metabolic disorders are characterized by impaired glucose and lipid metabolism, insulin resistance, and adipose tissue dysfunction. Interferon regulatory factor 1 (IRF1) is a transcription factor primarily involved in immune regulation; however, its role in adipocyte metabolic regulation remains incompletely understood. In this study, adipocyte-specific *Irf1* knockout (*Irf1* AKO) mice were generated using the Cre/loxP system and subjected to either a regular chow diet or a high-fat diet (HFD). Metabolic phenotyping, insulin signaling analysis, mitochondrial homeostasis-related assessment, and in vitro adipocyte experiments were performed. Adipocyte-specific IRF1 deficiency improved insulin-stimulated AKT phosphorylation in white adipose tissues and enhanced glucose tolerance and insulin sensitivity under HFD conditions. These metabolic improvements were accompanied by reduced oxygen consumption, energy expenditure, heat production, β3-adrenergic-induced lipolytic response, and cold tolerance. At the molecular level, IRF1 deficiency was associated with reduced TOMM20 expression, decreased mtDNA content, downregulation of oxidative phosphorylation-related genes, and reduced ATP levels in adipose tissues, suggesting altered mitochondrial homeostasis. In 3T3-L1 adipocytes, IRF1 knockdown increased insulin-stimulated AKT activation, glucose uptake, and lipid accumulation, whereas IRF1 overexpression showed opposite trends. Collectively, these findings suggest that adipocyte IRF1 is associated with insulin signaling, lipid metabolic remodeling, and mitochondrial homeostasis, and highlight a potential dissociation between improved insulin responsiveness and reduced energy expenditure in diet-induced obesity.

## 1. Introduction

Obesity has become a global epidemic and is strongly associated with metabolic disorders, including insulin resistance, type 2 diabetes mellitus, metabolic dysfunction-associated fatty liver disease and cardiovascular disease [1,2,3,4]. Excess nutrient intake and chronic energy imbalance promote adipose tissue expansion and dysfunction, which contribute to systemic metabolic disorders [5,6].

Adipose tissue is not only an energy storage organ but also an active endocrine organ that regulates whole-body glucose and lipid metabolism through adipokine secretion and inter-organ communication [7,8,9]. White adipocytes primarily store excess energy as triglycerides, whereas brown and beige adipocytes dissipate energy through mitochondrial thermogenesis [10,11,12]. In obesity, adipose tissue dysfunction is commonly associated with chronic low-grade inflammation, impaired insulin responsiveness, altered lipid turnover, and mitochondrial abnormalities [13,14,15].

Insulin signaling in adipocytes is primarily mediated by the insulin receptor substrate–PI3K–AKT pathway, which regulates glucose uptake, lipid metabolism, and metabolic homeostasis [16,17]. Impaired AKT activation in adipose tissue contributes to systemic insulin resistance and metabolic dysregulation [18,19]. Conversely, improvement of adipocyte insulin sensitivity can enhance glucose disposal and reduce circulating non-esterified fatty acids without necessarily reducing adipose tissue mass [20,21].

Mitochondria are essential organelles for adipocyte energy metabolism and thermogenic function. In white adipocytes, mitochondria participate in lipid handling, redox balance, and metabolic flexibility, whereas in brown and beige adipocytes, mitochondrial oxidative metabolism is central to adaptive thermogenesis and energy expenditure [22,23,24]. Mitochondrial biogenesis, oxidative phosphorylation, mtDNA maintenance, and ATP production are therefore critical for preserving adipocyte metabolic homeostasis [25,26]. TOMM20, a receptor component of the translocase of the outer mitochondrial membrane complex, is commonly used as a mitochondrial marker reflecting mitochondrial abundance and integrity [27]. In obesity, adipocyte mitochondrial dysfunction is associated with impaired oxidative capacity, altered lipid metabolism, inflammation, and insulin resistance, thereby contributing to obesity-related metabolic disorders [28,29].

Interferon regulatory factor 1 (IRF1) is a classical transcription factor originally identified as a regulator of interferon-responsive genes and is widely involved in antiviral defense, inflammatory responses, and immune regulation [30,31]. Beyond their canonical immune functions, IRF family members have recently emerged as important regulators of metabolic homeostasis [32]. In adipocytes, IRF4 regulates lipid handling, lipolysis, and thermogenic gene programs, whereas IRF3 and IRF7 have been implicated in adipose inflammation, insulin resistance, and diet-induced metabolic dysfunction [33,34,35,36,37]. In human adipocytes, IRF1 activation promotes inflammatory phenotypes associated with metabolic disease, suggesting a potential role for IRF1 in adipocyte dysfunction [38]. However, the metabolic role of IRF1 appears to be context-dependent, and its adipocyte-specific function in regulating insulin signaling, lipid metabolic remodeling, and mitochondrial homeostasis remains insufficiently understood.

We hypothesized that adipocyte IRF1 may contribute to metabolic homeostasis through its association with insulin signaling and mitochondrial homeostasis. To test this hypothesis, we generated adipocyte-specific *Irf1* knockout mice and combined in vivo metabolic phenotyping with in vitro adipocyte models to investigate the role of IRF1 in insulin signaling, lipid metabolic remodeling, energy expenditure, and mitochondrial homeostasis during obesity-induced metabolic dysfunction.

## 2. Results

### 2.1. Generation and Metabolic Characterization of Adipocyte-Specific Irf1 Knockout Mice

**Figure 1:**
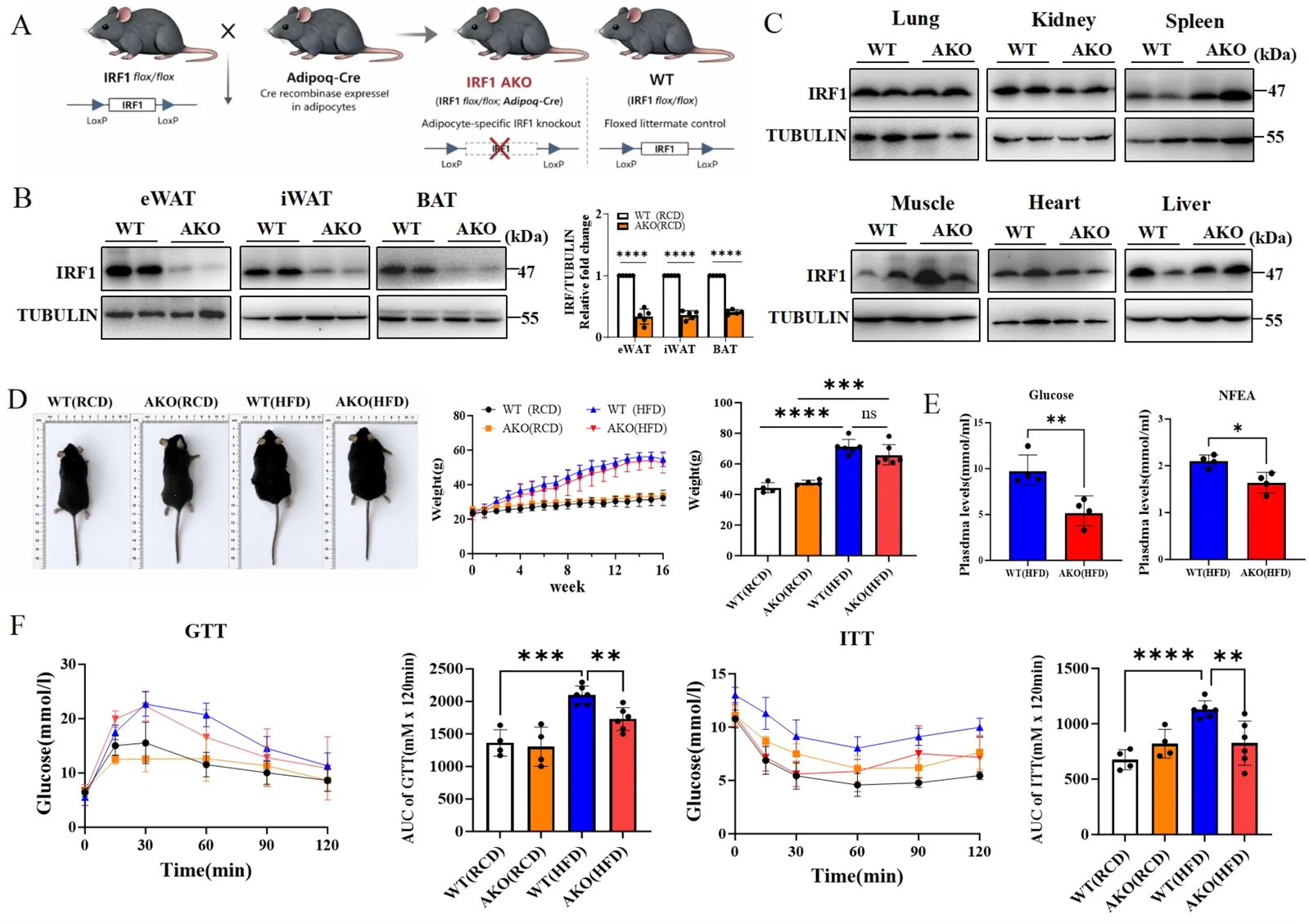
Generation and metabolic characterization of adipocyte-specific *Irf1* knockout mice. (**A**) Schematic diagram of adipocyte-specific *Irf1* knockout mouse generation using the Cre/loxP system. (**B**) Western blot analysis of IRF1 expression in adipose tissues, including eWAT, iWAT, and BAT. (**C**) Western blot analysis of IRF1 expression in non-adipose tissues, including lung, kidney, spleen, skeletal muscle, heart, and liver. (**D**) Representative images, body weight curves, and final body weight of WT and *Irf1* AKO mice under RCD and HFD conditions. (**E**) Fasting plasma glucose and NEFA levels after overnight fasting (12 h) in HFD-fed WT and *Irf1* AKO mice. (**F**) Glucose tolerance test (GTT) after overnight fasting (12 h) and insulin tolerance test (ITT) after 4–6 h fasting, with corresponding AUC analyses, in WT and *Irf1* AKO mice under RCD and HFD conditions. Data are presented as mean ± SEM. * *p* < 0.05, ** *p* < 0.01, *** *p* < 0.001, **** *p* < 0.0001; ns, not significant, *n* = 4–6.

To investigate the role of IRF1 in adipocyte metabolic regulation, adipocyte-specific *Irf1* knockout (*Irf1* AKO) mice were generated using the Cre/loxP system by crossing *Irf1*^flox/flox^ mice with Adipoq-Cre mice (Figure 1A). Western blot analysis showed that IRF1 protein expression was markedly reduced in adipose tissues, including eWAT, iWAT, and BAT, whereas IRF1 expression was not obviously altered in non-adipose tissues such as lung, kidney, spleen, skeletal muscle, heart, and liver (Figure 1B,C). These results confirmed efficient and adipose tissue-specific knockout of *Irf1*. WT and *Irf1* AKO mice were then subjected to RCD or HFD feeding to assess metabolic phenotypes. Under both dietary conditions, WT and *Irf1* AKO mice showed comparable gross morphology, body weight gain, and final body weight (Figure 1D). Under HFD conditions, fasting plasma glucose and NEFA levels were significantly reduced in *Irf1* AKO mice compared with WT controls (Figure 1E). GTT and ITT further showed improved glucose tolerance and enhanced insulin sensitivity in HFD-fed *Irf1* AKO mice, as indicated by reduced AUC values (Figure 1F). Additional plasma lipid parameters and PTT results are shown in Supplementary Figures S1 and S2. Together, these results suggest that adipocyte-specific IRF1 deficiency is associated with enhanced insulin signaling and improved glucose tolerance under HFD conditions without significantly affecting body weight.

### 2.2. IRF1 Deficiency Is Associated with Depot-Specific Adipose Tissue Remodeling Without Altering Systemic Fat Distribution

**Figure 2:**
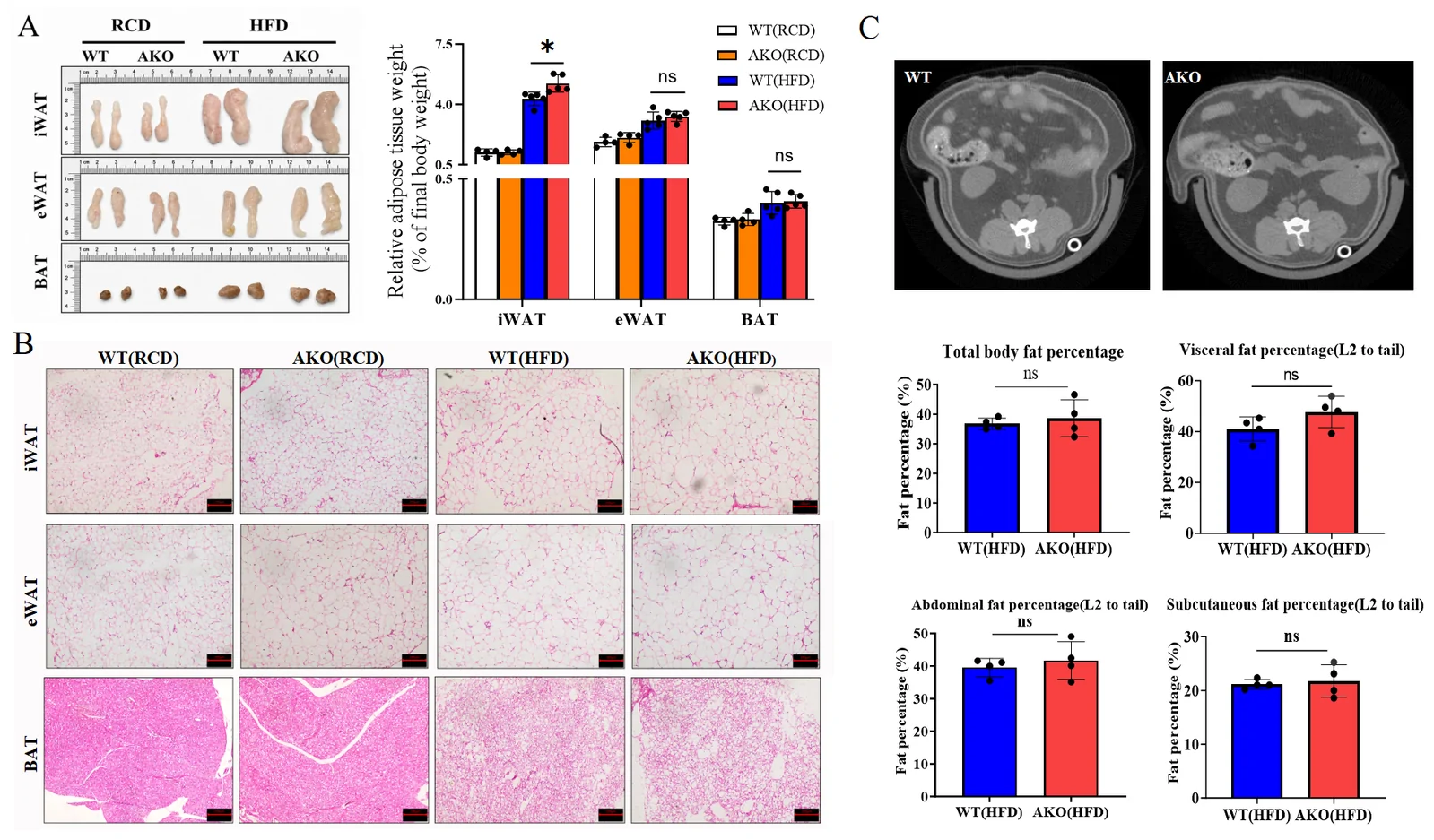
IRF1 deficiency is associated with depot-specific adipose tissue remodeling without affecting fat distribution. (**A**) Representative images and relative weights of iWAT, eWAT, and BAT normalized to final body weight in WT and *Irf1* AKO mice under RCD and HFD conditions. (**B**) Representative H&E staining images of iWAT, eWAT, and BAT. (scale bar = 200 μm). Quantitative analysis of adipocyte area is shown in Supplementary Figure S4. (**C**) Representative Micro-CT images and quantification of total body fat, visceral fat, abdominal fat, and subcutaneous fat percentages in HFD-fed WT and *Irf1* AKO mice. Values are mean ± SEM. ns, not significant; * *p* < 0.05, *n* = 4–5.

To evaluate the effect of IRF1 deficiency on adipose tissue expansion and fat distribution, adipose tissue mass, histology, and body fat composition were analyzed in WT and *Irf1* AKO mice under RCD and HFD conditions. Under HFD conditions, *Irf1* AKO mice exhibited a modest but significant increase in relative iWAT weight normalized to final body weight, whereas eWAT and BAT weights were not significantly altered (Figure 2A). Histological analysis showed mild morphological changes and a tendency toward increased adipocyte size in iWAT of HFD-fed *Irf1* AKO mice, while no obvious morphological changes were observed in eWAT or BAT (Figure 2B). Quantitative analysis of adipocyte area was performed using ImageJ (version 1.54, National Institutes of Health, Bethesda, MD, USA) and is presented in Supplementary Figure S4. Micro-CT analysis further showed no significant differences in total body fat, visceral fat, abdominal fat, or subcutaneous fat percentages between HFD-fed WT and *Irf1* AKO mice (Figure 2C). Together, these results suggest that adipocyte-specific IRF1 deficiency is associated with depot-specific remodeling of iWAT without markedly altering systemic fat distribution.

### 2.3. IRF1 Deficiency Enhances Insulin-Stimulated AKT Signaling in White Adipose Tissues

**Figure 3:**
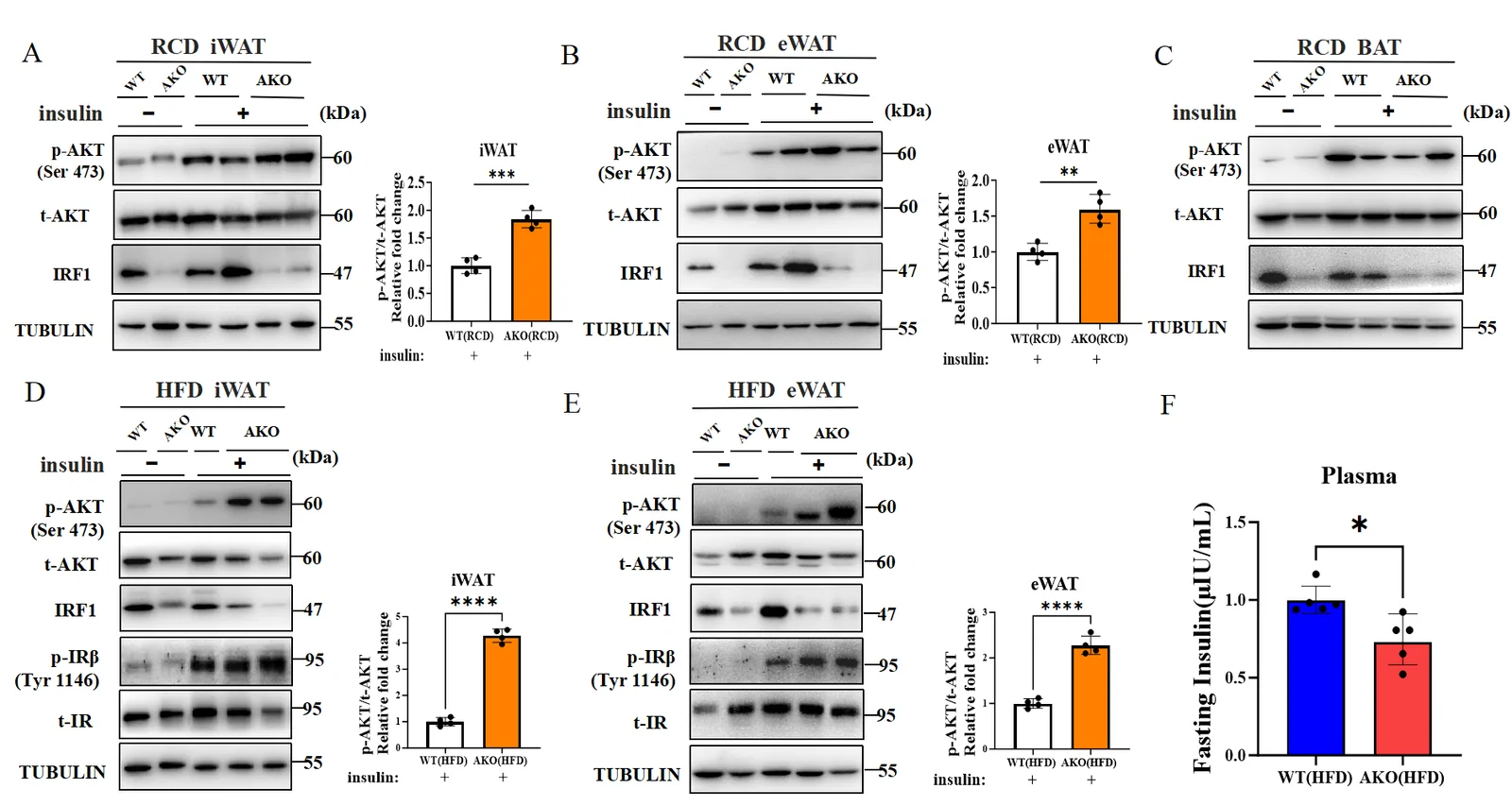
IRF1 deficiency enhances insulin-stimulated AKT phosphorylation in white adipose tissues. (**A**–**C**) Western blot analysis of p-AKT, total AKT, IRF1, and TUBULIN in iWAT, eWAT, and BAT under RCD conditions after insulin stimulation following 4–6 h fasting. (**D**,**E**) Western blot analysis of p-AKT, total AKT, IRF1, p-IRβ, total IR, and TUBULIN in iWAT and eWAT under HFD conditions after insulin stimulation following 4–6 h fasting. (**F**) Fasting plasma insulin levels measured after overnight fasting (12 h) in HFD-fed WT and *Irf1* AKO mice. Data are presented as mean ± SEM. * *p* < 0.05, ** *p* < 0.01, *** *p* < 0.001, **** *p* < 0.0001, *n* = 4–5.

Insulin signaling activity was evaluated by assessing AKT phosphorylation in adipose tissues after acute insulin stimulation. Under RCD conditions, *Irf1* AKO mice showed increased insulin-stimulated AKT phosphorylation in iWAT and eWAT, whereas the response in BAT was relatively modest (Figure 3A–C). Under HFD conditions, insulin-stimulated AKT phosphorylation was markedly enhanced in both iWAT and eWAT of *Irf1* AKO mice, accompanied by an apparent increase in IRβ phosphorylation (Figure 3D,E). Consistently, fasting plasma insulin levels were significantly reduced in HFD-fed *Irf1* AKO mice compared with WT controls (Figure 3F). Together, these results suggest that adipocyte-specific IRF1 deficiency enhances insulin-stimulated AKT signaling in white adipose tissues and improves systemic insulin responsiveness under HFD conditions.

### 2.4. IRF1 Deficiency Is Associated with Reduced Energy Expenditure, Lipolytic Response, and Cold-Exposure Response

**Figure 4:**
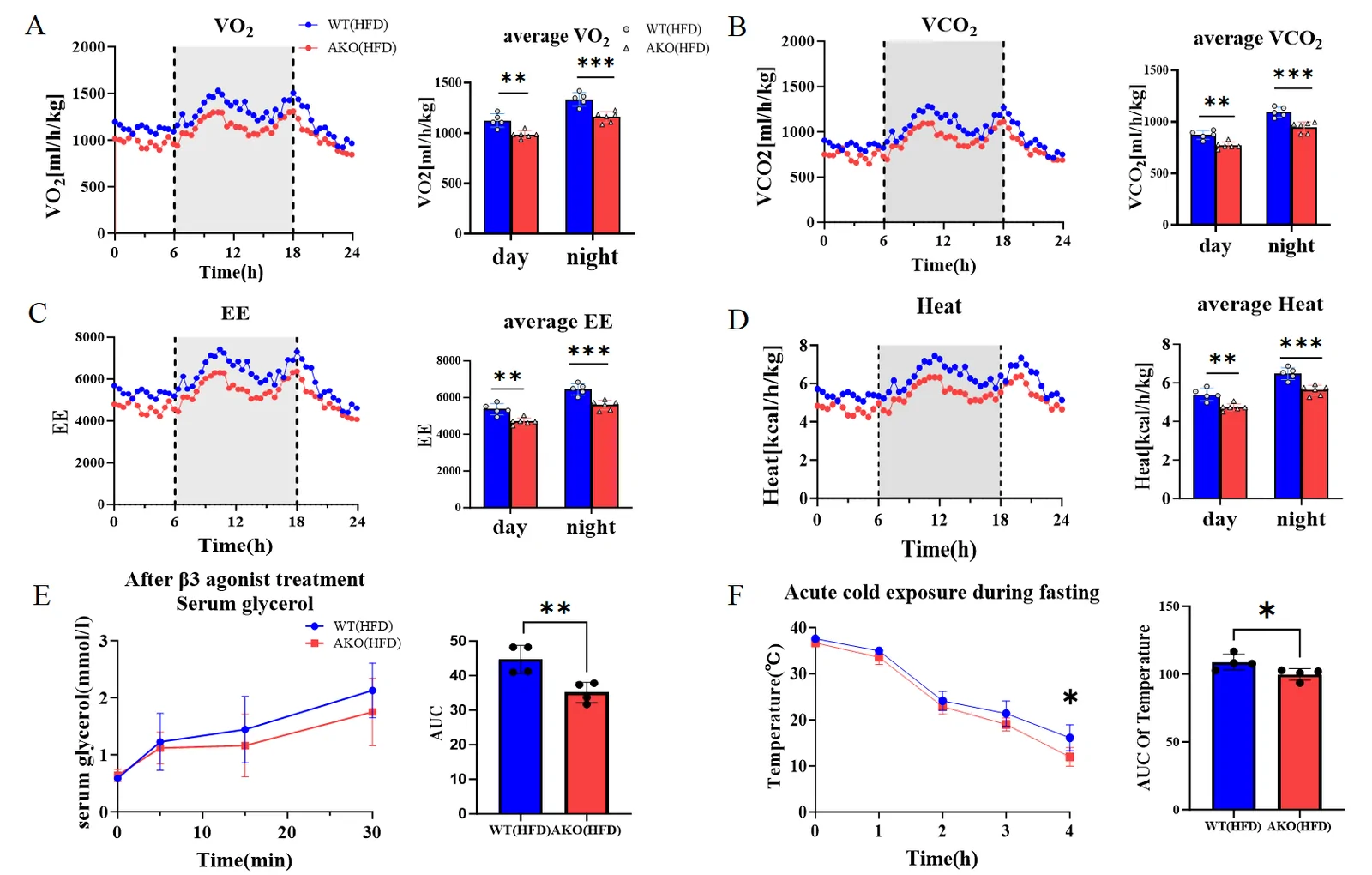
IRF1 deficiency is associated with reduced energy expenditure, lipolytic response, and cold-exposure response. (**A**–**D**) Lean mass-normalized VO_2_, VCO_2_, energy expenditure (EE), and heat production in HFD-fed WT and *Irf1* AKO mice, with average values during light and dark phases. (**E**) Serum glycerol levels after β3-adrenergic agonist stimulation following 4 h fasting. AUC analysis was performed to evaluate the overall glycerol response over the stimulation period. (**F**) Body temperature changes during acute cold exposure after 4 h fasting. AUC analysis was performed to evaluate the overall body temperature maintenance response during cold exposure. Independent cohorts of HFD-fed WT and *Irf1* AKO mice were used for the metabolic cage analysis, β3-adrenergic stimulation, and cold exposure experiments after 16 weeks of HFD feeding. Data are presented as mean ± SEM. * *p* < 0.05, ** *p* < 0.01, *** *p* < 0.001, *n* = 4–6.

To evaluate the role of IRF1 in systemic energy metabolism, HFD-fed WT and *Irf1* AKO mice were analyzed by metabolic cage assessment, β3-adrenergic stimulation, and acute cold exposure. *Irf1* AKO mice exhibited significantly reduced oxygen consumption (VO_2_), carbon dioxide production (VCO_2_), energy expenditure (EE), and heat production during both light and dark phases (Figure 4A–D). Following β3-adrenergic agonist stimulation, *Irf1* AKO mice showed a reduced cumulative glycerol response over the stimulation period, as reflected by decreased AUC values (Figure 4E). During acute cold exposure after 4 h fasting, *Irf1* AKO mice showed a tendency toward reduced body temperature maintenance, suggesting an altered adaptive thermogenic response under cold stress (Figure 4F). RER, food intake, water intake, and locomotor activity are shown in Supplementary Figure S3. Together, these results suggest that adipocyte-specific IRF1 deficiency is associated with reduced systemic energy expenditure, attenuated β3-adrenergic-induced lipolytic response, and altered cold-exposure response under HFD conditions.

### 2.5. IRF1 Deficiency Induces Depot-Specific Metabolic Remodeling in Adipose Tissues Under HFD Conditions

**Figure 5:**
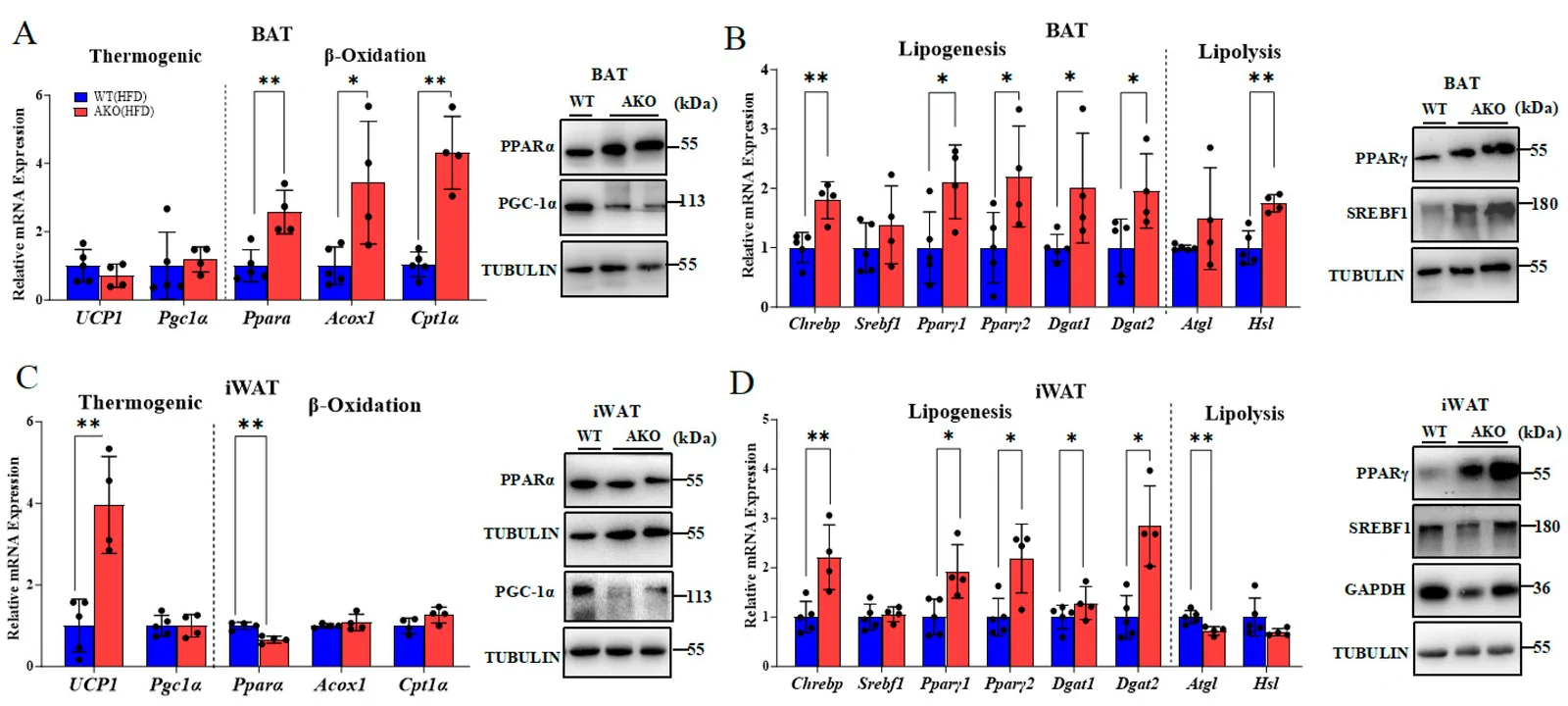
IRF1 deficiency induces depot-specific lipid metabolic reprogramming in adipose tissues under high-fat diet. (**A**) Gene expression of thermogenic and fatty acid oxidation-related markers, together with protein levels of PPARα and PGC-1α in brown adipose tissue (BAT) from HFD-fed WT and *Irf1* AKO mice. (**B**) Gene expression of lipogenesis- and lipolysis-related markers, together with protein levels of PPARγ and SREBF1 in BAT. (**C**) Gene expression of thermogenic and fatty acid oxidation-related markers, together with protein levels of PPARα and PGC-1α in inguinal white adipose tissue (iWAT). (**D**) Gene expression of lipogenesis- and lipolysis-related markers, together with protein levels of PPARγ and SREBF1 in iWAT. Data are presented as mean ± SEM. * *p* < 0.05, ** *p* < 0.01, *n* = 4–5.

To determine whether IRF1 deficiency affects adipose tissue metabolic programs under HFD conditions, genes and proteins related to thermogenesis, fatty acid oxidation, lipogenesis, and lipolysis were examined in BAT and iWAT. In BAT, *Irf1* AKO mice showed increased expression of fatty acid oxidation-related genes, including *Ppara*, *Acox1*, and *Cpt1a*, whereas the thermogenic genes *Ucp1* and *Pgc1a* were not significantly altered. Consistently, PPARα protein expression was increased. In addition, genes involved in lipogenesis and lipolysis, including *Chrebp*, *Pparg1*, *Pparg2*, *Dgat1*, *Dgat2*, and *Hsl*, were upregulated, accompanied by increased PPARγ and SREBF1 protein expression (Figure 5A,B). The concurrent elevation of lipogenic and lipolytic gene expression in BAT may reflect enhanced lipid metabolic turnover or compensatory metabolic remodeling rather than a simple increase in a single lipid metabolic pathway. In iWAT, *Irf1* AKO mice exhibited increased *Ucp1* expression, while *Pgc1a* remained unchanged. In contrast to BAT, *Ppara* expression was reduced, whereas *Acox1* and *Cpt1a* were not markedly changed. Lipogenic genes, including *Chrebp*, *Pparg1*, *Pparg2*, *Dgat1*, and *Dgat2*, were increased, while lipolysis-related genes showed an overall downward trend, particularly *Atgl*. Western blot analysis further showed increased PPARγ protein expression in iWAT (Figure 5C,D). In contrast, eWAT showed no marked changes in lipid metabolic gene expression or TOMM20 protein expression under HFD conditions (Supplementary Figure S5). Together, these results suggest that adipocyte-specific IRF1 deficiency is associated with depot-specific metabolic remodeling under HFD conditions, with more evident changes in BAT and iWAT than in eWAT.

### 2.6. IRF1 Deficiency Alters Mitochondrial Homeostasis in Adipose Tissues

**Figure 6:**
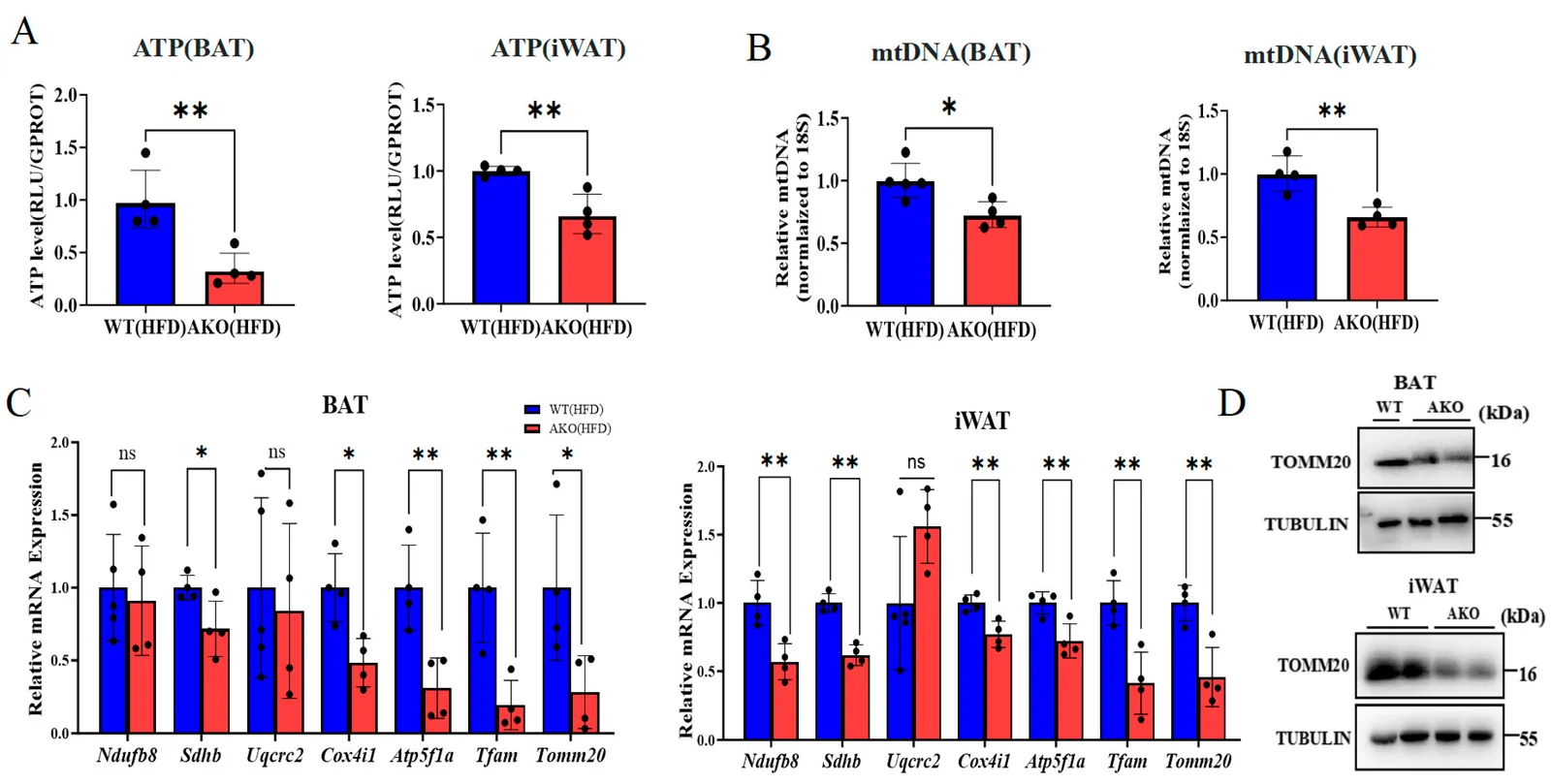
IRF1 deficiency is associated with altered mitochondrial homeostasis in adipose tissues under HFD conditions. (**A**) ATP levels in BAT and iWAT from WT and *Irf1* AKO mice under high-fat diet (HFD). (**B**) Relative mtDNA content in BAT and iWAT. (**C**) mRNA expression of mitochondrial OXPHOS-related genes (*Ndufb8*, *Sdhb*, *Uqcrc2*, *Cox4i1*, *Atp5f1a*, *Tfam*, *Tomm20*) in BAT and iWAT. (**D**) Representative Western blot and quantification of TOMM20 protein levels in BAT and iWAT. Data are presented as mean ± SEM. ns, not significant; * *p* < 0.05, ** *p* < 0.01, *n* = 4–5.

To determine whether IRF1 deficiency affects mitochondrial homeostasis in adipose tissues, ATP levels, mtDNA content, mitochondrial oxidative phosphorylation-related gene expression, and TOMM20 protein levels were examined in BAT and iWAT from HFD-fed WT and *Irf1* AKO mice. *Irf1* AKO mice showed significantly reduced ATP levels in both BAT and iWAT. Consistently, mtDNA content was also decreased in these two adipose depots, suggesting reduced mitochondrial content and altered mitochondrial metabolic status (Figure 6A,B). Further analysis showed that multiple mitochondrial oxidative phosphorylation-related genes were downregulated in BAT and iWAT of *Irf1* AKO mice, including Sdhb, Cox4i1, Atp5f1a, Tfam, and *Tomm20*, with depot-specific differences in individual genes. In line with the mRNA results, TOMM20 protein expression was markedly reduced in both BAT and iWAT (Figure 6C,D). Together, these results suggest that adipocyte-specific IRF1 deficiency is associated with altered mitochondrial homeostasis in adipose tissues under HFD conditions.

### 2.7. IRF1 Knockdown Enhances Insulin Responsiveness and Lipid Accumulation While Impairing Mitochondrial Homeostasis in Adipocytes

**Figure 7:**
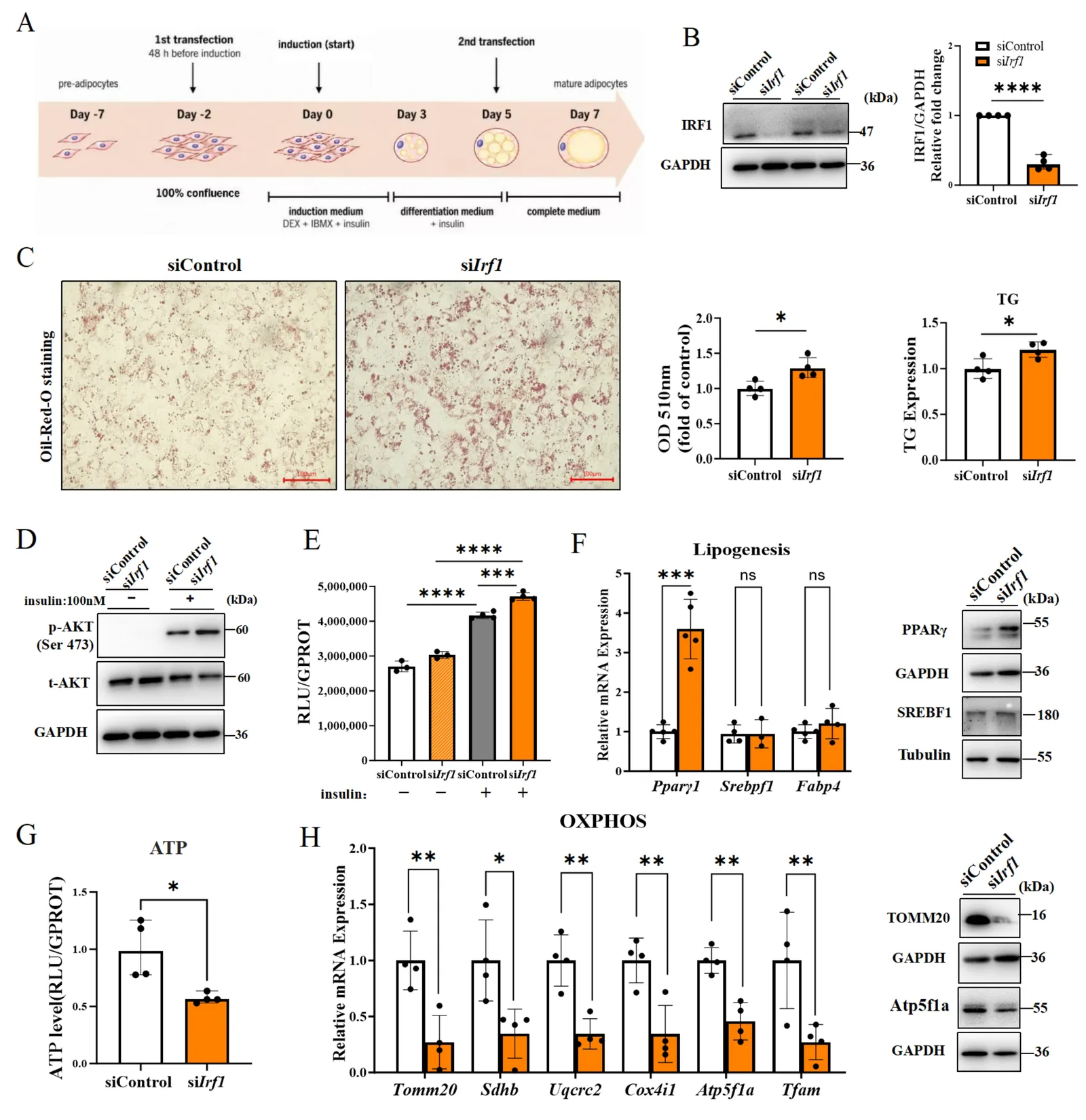
IRF1 knockdown enhances insulin responsiveness and lipid accumulation but impairs mitochondrial function in 3T3-L1 adipocytes. (**A**) Schematic representation of the siRNA-mediated *Irf1* knockdown strategy during 3T3-L1 adipocyte differentiation. (**B**) Western blot validation and densitometric quantification of IRF1 protein expression in siControl and si*Irf1* adipocytes. (**C**) Oil Red O staining, spectrophotometric quantification of Oil Red O extraction at 510 nm, and intracellular triglyceride content in differentiated adipocytes. Scale bar, 100 μm. (**D**) Western blot analysis of AKT phosphorylation at Ser473 under basal and insulin-stimulated conditions. (**E**) Glucose uptake assay in siControl and si*Irf1* adipocytes in the absence or presence of insulin stimulation. (**F**) mRNA expression of adipogenic and lipogenic markers, together with representative Western blot analysis of PPARγ and SREBF1. (**G**) Intracellular ATP levels in siControl and si*Irf1* adipocytes. (**H**) mRNA expression of mitochondrial oxidative phosphorylation-related genes, together with representative Western blot analysis of TOMM20 and ATP5F1A. Data are presented as mean ± SEM. * *p* < 0.05, ** *p* < 0.01, *** *p* < 0.001, **** *p* < 0.0001, *n* = 4–5.

To further determine whether IRF1 regulates adipocyte metabolism in a cell-autonomous manner, IRF1 was knocked down in 3T3-L1 adipocytes during differentiation using a two-step siRNA transfection strategy. Western blot analysis confirmed an efficient reduction in IRF1 protein expression in si*Irf1* adipocytes (Figure 7A,B). IRF1 knockdown increased lipid accumulation, as shown by enhanced Oil Red O staining, increased Oil Red O absorbance at 510 nm, and elevated intracellular triglyceride levels. In parallel, insulin-stimulated AKT phosphorylation was enhanced in si*Irf1* adipocytes. The glucose uptake assay further showed increased glucose uptake in IRF1-knockdown adipocytes under insulin-stimulated conditions, indicating enhanced insulin responsiveness (Figure 7C–E). At the molecular level, IRF1 knockdown increased the expression of the adipogenic marker *Pparg1*, whereas *Srebf1* and *Fabp4* were not significantly altered. Consistently, PPARγ protein expression was increased, while SREBF1 showed no obvious elevation (Figure 7F). In contrast, mitochondrial homeostasis was impaired, as evidenced by reduced ATP levels. Moreover, multiple mitochondrial oxidative phosphorylation-related genes, including *Tomm20*, *Sdhb*, *Uqcrc2*, *Cox4i1*, *Atp5f1a*, and *Tfam*, were downregulated in si*Irf1* adipocytes. Consistently, TOMM20 and ATP5F1A protein levels were also reduced (Figure 7G,H). Together, these results suggest that IRF1 knockdown promotes lipid accumulation and insulin responsiveness while impairing mitochondrial homeostasis in adipocytes.

### 2.8. IRF1 Gain-of-Function Exerts Opposite Metabolic Effects to IRF1 Knockdown in 3T3-L1 Adipocytes

**Figure 8:**
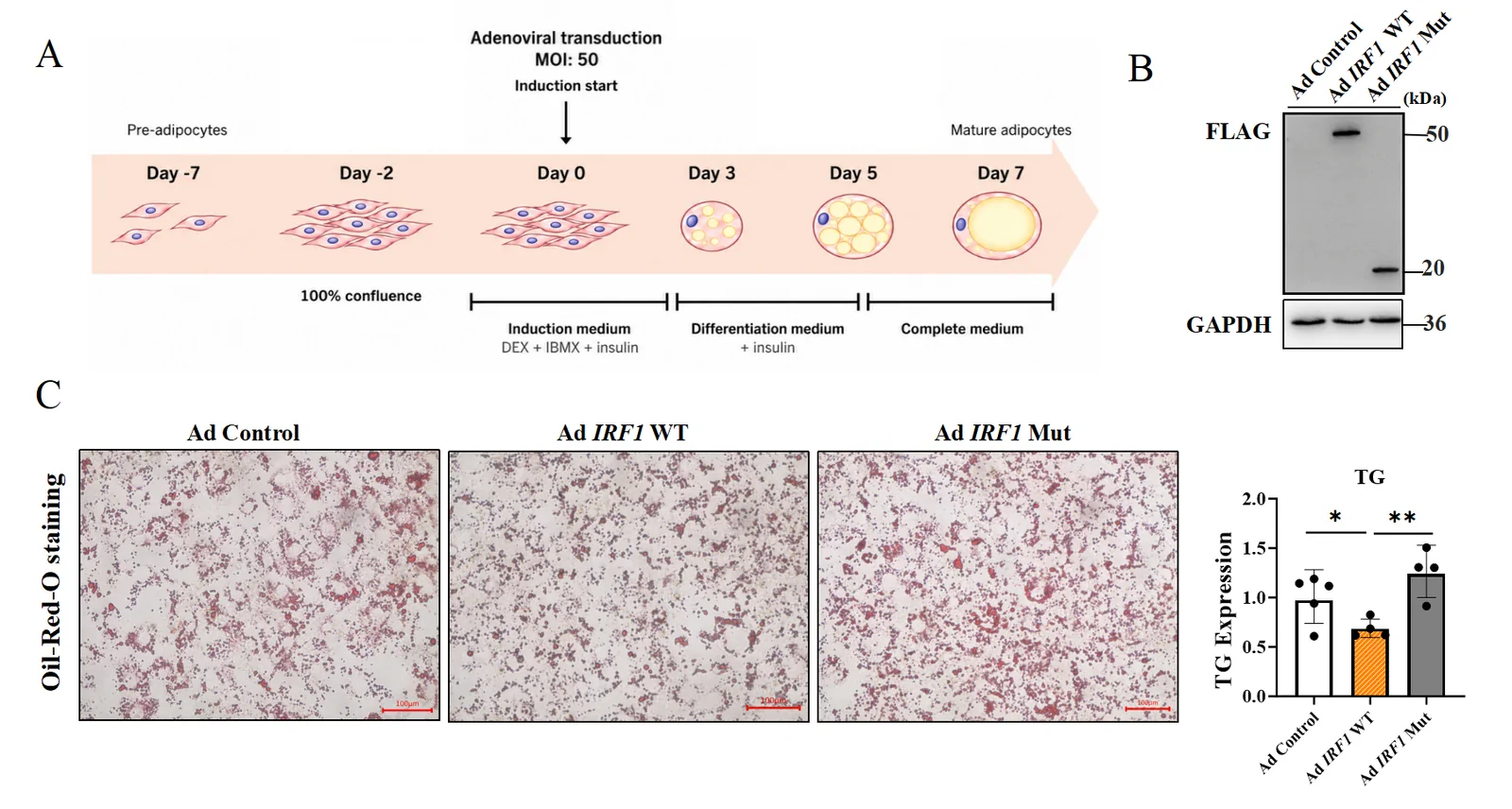

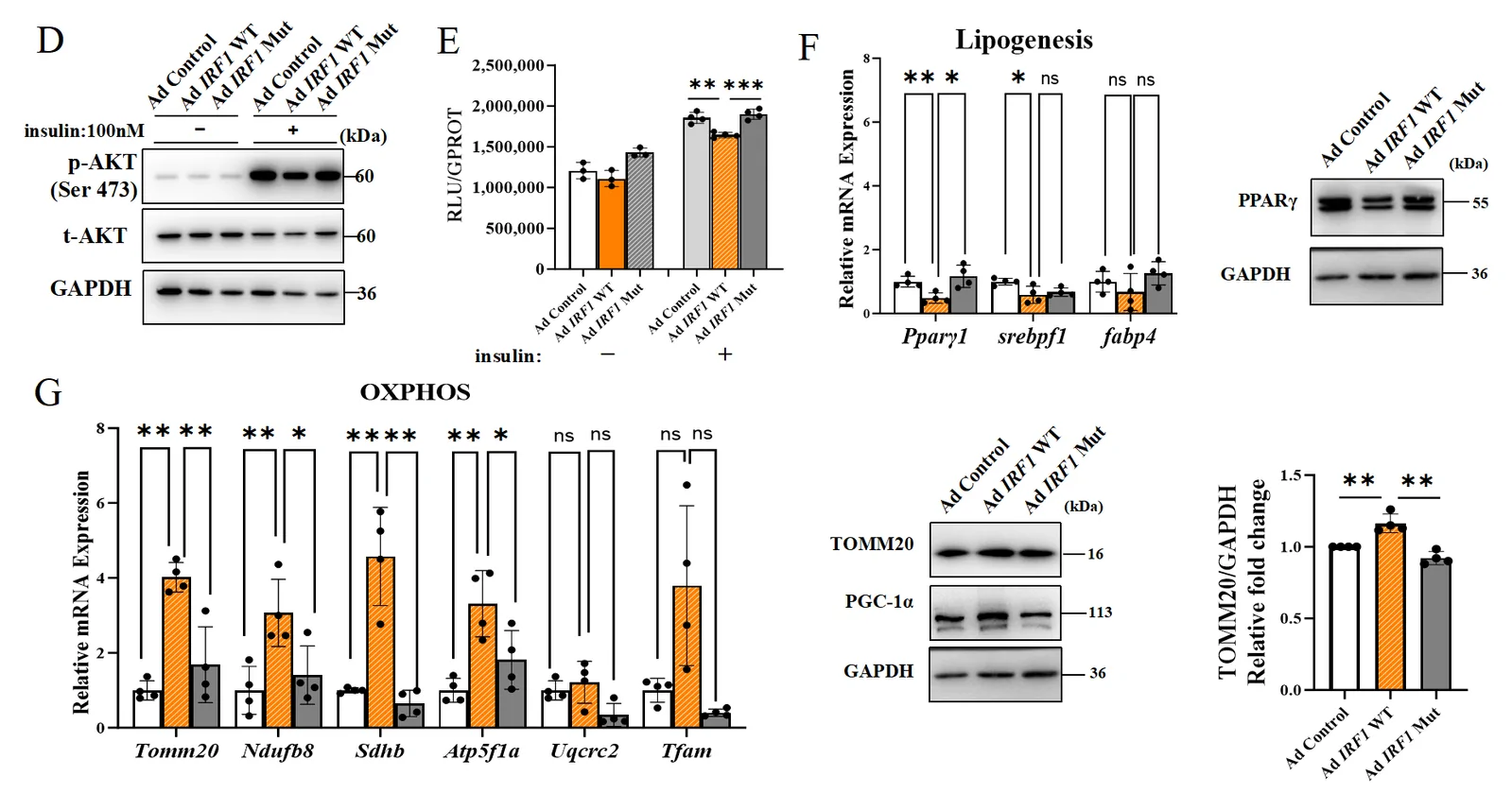
IRF1 gain-of-function exerts opposite metabolic effects to IRF1 knockdown in 3T3-L1 adipocytes. (**A**) Schematic representation of adenoviral IRF1 expression during 3T3-L1 adipocyte differentiation. (**B**) Western blot analysis of FLAG-tagged full-length IRF1 WT and truncated IRF1 Mut expression in Ad Control-, Ad IRF1 WT-, and Ad IRF1 Mut-infected adipocytes. (**C**) Oil Red O staining and intracellular triglyceride levels in differentiated adipocytes. Scale bar, 100 μm. (**D**) Western blot analysis of AKT phosphorylation at Ser473 under basal and insulin-stimulated conditions. (**E**) Glucose uptake assay in Ad Control, Ad IRF1 WT, and Ad IRF1 Mut adipocytes with or without insulin stimulation. (**F**) mRNA expression of lipogenic/adipogenic markers, together with representative Western blot analysis of PPARγ. (**G**) mRNA expression of mitochondrial OXPHOS-related genes, together with representative Western blot analysis of TOMM20 and PGC-1α. Data are presented as mean ± SEM. * *p* < 0.05, ** *p* < 0.01, *** *p* < 0.001; ns, not significant.

To further complement the IRF1 knockdown experiments, gain-of-function assays were performed in 3T3-L1 adipocytes using adenoviruses expressing FLAG-tagged full-length IRF1 wild-type protein or truncated dominant-negative IRF1 mutant protein during differentiation (Figure 8A). Western blot analysis using an anti-FLAG antibody confirmed the expression of full-length IRF1 WT and truncated IRF1 Mut proteins in adenovirus-infected adipocytes (Figure 8B). Compared with Ad Control cells, Ad IRF1 WT reduced lipid accumulation, as shown by decreased Oil Red O staining and intracellular triglyceride levels. In contrast, Ad IRF1 Mut showed an opposite effect, with increased triglyceride accumulation compared with Ad IRF1 WT, suggesting that the truncated mutant attenuated the lipid-suppressive effect of IRF1 (Figure 8C). Consistent with the opposite pattern observed in si*Irf1* adipocytes, Ad IRF1 WT reduced insulin-stimulated AKT phosphorylation and glucose uptake, whereas Ad IRF1 Mut partially reversed these effects (Figure 8D,E). At the molecular level, Ad IRF1 WT decreased the expression of lipogenic/adipogenic markers, including *Pparg1* and *Srebf1*, together with reduced PPARγ protein expression (Figure 8F). In contrast, Ad IRF1 WT increased the expression of mitochondrial oxidative phosphorylation-related genes, including *Tomm20*, *Ndufb8*, *Sdhb*, and *Atp5f1a*. Consistently, TOMM20 protein expression was increased in Ad IRF1 WT adipocytes, whereas this effect was attenuated in Ad IRF1 Mut cells (Figure 8G). Together, these gain-of-function results complement the IRF1 knockdown data and further support that IRF1 restrains adipocyte lipid accumulation and insulin responsiveness while maintaining mitochondrial metabolic markers.

## 3. Discussion

Obesity-associated metabolic disorders are characterized by impaired insulin sensitivity, altered lipid metabolism, chronic low-grade inflammation, and dysfunctional energy homeostasis. During obesity, adipose tissue undergoes extensive remodeling, including adipocyte hypertrophy, extracellular matrix remodeling, inflammatory activation, and metabolic dysfunction [39,40]. In the present study, we investigated the adipocyte-specific role of IRF1 in metabolic regulation using adipocyte-specific *Irf1* knockout (*Irf1* AKO) mice combined with in vitro adipocyte models. Our findings suggest that IRF1 in adipocytes may be involved in insulin signaling, lipid metabolism, and mitochondrial homeostasis.

A major finding of this study is that adipocyte-specific IRF1 deficiency is associated with improved insulin sensitivity and glucose homeostasis under high-fat diet conditions, as evidenced by enhanced insulin-stimulated AKT phosphorylation in white adipose tissues and improved systemic glucose tolerance. These results suggest that IRF1 deficiency is associated with increased insulin responsiveness in adipocytes. Importantly, these metabolic improvements occurred without significant changes in body weight or systemic fat distribution, indicating that IRF1 deficiency mainly influences adipocyte functional properties rather than overall adiposity. These observations are consistent with previous studies demonstrating that adipose tissue insulin action, fatty acid flux, and lipolytic regulation can influence systemic glucose homeostasis independently of major changes in fat mass [20,41]. Although previous studies have implicated IRF1 in adipocyte insulin responsiveness, the present study extends these observations by using an adipocyte-specific *Irf1* knockout mouse model under diet-induced obesity. Importantly, our findings suggest that improved insulin signaling after adipocyte IRF1 deficiency occurs together with reduced energy expenditure, attenuated lipolytic response, and altered mitochondrial homeostasis-related markers. Therefore, this study highlights a potential dissociation between adipocyte insulin responsiveness and energy dissipation in the absence of IRF1.

Despite improved insulin signaling, *Irf1* AKO mice exhibited reduced energy expenditure, attenuated β3-adrenergic-induced lipolytic response, and altered cold-exposure response, indicating a dissociation between glucose metabolic improvement and energy dissipation. Mitochondrial oxidative metabolism is essential for adipocyte thermogenesis and systemic energy expenditure [11,42,43]. In parallel, β3-adrenergic–driven lipolysis supplies fatty acids for mitochondrial oxidation and heat production [44,45]. Accordingly, an attenuated lipolytic response may limit substrate availability for thermogenesis and contribute to altered cold-exposure adaptation. Consistent with this, disruption of adipose lipolysis or mitochondrial homeostasis has been shown to uncouple insulin sensitivity from energy expenditure in metabolic disease models [20]. Together, these findings suggest that adipocyte IRF1 may be involved in the regulation of insulin responsiveness, lipid mobilization, and mitochondrial metabolic homeostasis.

At the molecular level, IRF1 deficiency is associated with depot-specific metabolic changes in adipose tissues. In brown adipose tissue, genes involved in fatty acid oxidation, lipid metabolism, and lipolytic pathways were broadly upregulated, suggesting an increased but potentially dysregulated metabolic state. In inguinal white adipose tissue, IRF1 deficiency increased *Ucp1* expression while simultaneously promoting lipogenic gene expression and reducing lipolysis-related gene expression, indicating a mixed anabolic and thermogenic remodeling phenotype. Previous studies have demonstrated that adipose tissue depots exhibit distinct transcriptional and metabolic programs in response to nutritional and thermogenic stimuli [46,47], and that UCP1-independent mechanisms and mitochondrial quality control also contribute substantially to thermogenic regulation [12,42]. Together, these findings suggest that IRF1 may not regulate a single metabolic pathway but instead may participate in coordinating multi-layered metabolic programs across adipose depots. Although lipolysis-related genes were altered in BAT, protein-level validation of key lipolytic enzymes, such as ATGL and phosphorylated HSL, was not performed in the present study. Therefore, these findings should be interpreted as changes in lipid metabolic programs rather than direct evidence of enhanced lipolytic activity.

Another important finding of this study is that IRF1 deficiency is associated with altered mitochondrial homeostasis-related changes in both BAT and iWAT. This is reflected by reduced ATP levels, decreased mitochondrial DNA content, and downregulation of oxidative phosphorylation-related genes, which collectively indicate altered mitochondrial status [48,49]. Consistently, TOMM20 expression was significantly reduced, suggesting reduced mitochondrial content and altered mitochondrial protein import capacity [50]. Together, these findings suggest that IRF1 may contribute to the maintenance of mitochondrial homeostasis in adipocytes.

Importantly, altered mitochondrial homeostasis may contribute to the reduced thermogenic adaptation observed in *Irf1* AKO mice. Although the classical thermogenic marker *Ucp1* was not uniformly reduced, mitochondrial gene expression and ATP-generating capacity are markedly suppressed, suggesting that the altered cold-exposure response may involve changes in mitochondrial content and metabolic capacity rather than UCP1 abundance alone [51,52]. This is consistent with previous studies highlighting the importance of mitochondrial quality control in regulating adaptive thermogenesis in adipose tissue [53,54]. In particular, disruption of mitochondrial dynamics or protein import has been shown to uncouple thermogenic gene expression from actual energy expenditure [55,56].

Notably, reduced TOMM20 expression may reflect altered mitochondrial protein import and mitochondrial abundance. Previous studies have shown that mitochondrial outer membrane import machinery is essential for mitochondrial remodeling and thermogenic adaptation [56]. In models of thermogenic dysfunction, decreased TOMM20 has been associated with reduced PGC1α expression and altered oxidative phosphorylation-related pathways [57], whereas its expression positively correlates with cold-induced thermogenic activation.

Furthermore, emerging evidence suggests that mitochondrial protein import and quality control systems are central regulators of adipose tissue energy metabolism. Disruption of mitochondrial import machinery or mitophagy impairs brown adipose tissue function and adaptive thermogenesis [53,58,59]. Collectively, these findings support the concept that mitochondrial integrity, rather than single thermogenic gene expression, is a key determinant of adipose tissue energy dissipation capacity.

Taken together, our findings suggest that IRF1 is associated with adipocyte insulin signaling, lipid metabolic programming, and mitochondrial homeostasis. IRF1 deficiency enhances insulin sensitivity and glucose utilization while simultaneously altering mitochondrial homeostasis-related processes and reducing energy expenditure, suggesting a potential functional trade-off between glucose metabolic efficiency and energy dissipation in obesity.

However, several limitations should be acknowledged. First, whether IRF1 directly regulates mitochondrial-related genes such as *Tomm20* remains to be determined. Second, direct assessments of mitochondrial respiration, such as Seahorse metabolic flux analysis or high-resolution respirometry, were not performed in this study. Therefore, the mitochondrial findings should be interpreted as changes in mitochondrial homeostasis-related markers rather than direct measurements of mitochondrial respiratory function. Third, because IRF1 is closely linked to inflammatory regulation, inflammatory markers in adipose tissues were not systematically examined in the current adipocyte-specific *Irf1* knockout model. Future studies are needed to determine whether IRF1-mediated metabolic effects interact with adipose inflammatory pathways. In addition, future studies incorporating ChIP-seq, promoter activity assays, Seahorse metabolic flux analysis, and adipose tissue–specific rescue models will be valuable for further defining the molecular mechanisms underlying IRF1-associated metabolic regulation.

## 4. Materials and Methods

### 4.1. Animal Models and Diet Intervention

All animal procedures were approved by the Institutional Animal Care and Use Committee of Nanjing Medical University (IACUC-2209011). Adipocyte-specific *Irf1* knockout mice were generated by crossing *Irf1^flox/flox^* mice with Adipoq-Cre transgenic mice, and littermate *Irf1^flox/flox^* mice without Cre were used as wild-type controls. Male mice at 8 weeks of age were housed under specific pathogen-free conditions and maintained under a 12 h light/dark cycle with free access to food and water. After one week of acclimatization, mice were randomly assigned to either a regular chow diet (RCD, 10% kcal fat; Research Diets, Inc., New Brunswick, NJ, USA) or a high-fat diet (HFD, 60% kcal fat; Research Diets, Inc., New Brunswick, NJ, USA) for 16 weeks. Body weight was monitored throughout the experimental period. At the end of the experiment, mice were euthanized, and tissues including inguinal white adipose tissue (iWAT), epididymal white adipose tissue (eWAT), brown adipose tissue (BAT), liver, skeletal muscle, lung, kidney, and spleen were collected. Tissues were either snap-frozen for molecular analyses or fixed for histological examination. Adipose tissues were fixed in 10% neutral-buffered formalin, embedded in paraffin, sectioned at 5 μm, and stained with hematoxylin and eosin (H&E). Images were captured using a light microscope, and adipocyte morphology was analyzed using ImageJ software (version 1.54, National Institutes of Health, Bethesda, MD, USA).

### 4.2. Cell Culture and Adipogenic Differentiation

The Mouse embryonic fibroblast cell line 3T3-L1 was purchased from the American Type Culture Collection (ATCC, USA). 3T3-L1 preadipocytes were cultured in Dulbecco’s Modified Eagle Medium (DMEM) supplemented with 10% fetal bovine serum (FBS) at 37 °C in a humidified atmosphere containing 5% CO_2_. Adipogenic differentiation was induced at confluence (designated as day 0). Cells were cultured in DMEM containing insulin (1 μg/mL), IBMX (0.5 mM), and dexamethasone (1 μM) for 48 h. Subsequently, cells were maintained in DMEM supplemented with insulin until day 7–8, at which point mature adipocytes were obtained for further experiments.

### 4.3. Transfection of siRNA and Infection of Adenovirus

3T3-L1 adipocytes were transfected with siRNA using Lipofectamine 2000 (Thermo Fisher Scientific, Waltham, MA, USA)according to the manufacturer’s instructions. Mouse *Irf1* siRNA (sc-35707, Santa Cruz Biotechnology, Dallas, TX, USA) was used for gene silencing experiments. Adenoviruses encoding full-length human IRF1 or a truncated dominant-negative IRF1 were purchased from Genechem (Shanghai, China) and used for overexpression or functional inhibition in 3T3-L1 adipocytes.

### 4.4. Glucose Tolerance Test, Insulin Tolerance Test and Pyruvate Tolerance Test

Glucose tolerance test (GTT), insulin tolerance test (ITT) and pyruvate tolerance test (PTT) were performed after dietary intervention. For GTT, mice were fasted for 12–16 h and intraperitoneally injected with glucose solution (Kelong Pharmaceutical Co., Ltd., Shijiazhuang, China) at a dose of 1.0 g/kg body weight. For ITT, mice were fasted for 4–6 h and intraperitoneally injected with insulin (Jiangsu Wanbang Biopharmaceutical Group Co., Ltd., Xuzhou, China) at a dose of 0.75 U/kg body weight. For PTT, mice were fasted for 12–14 h and intraperitoneally injected with sodium pyruvate (Sigma-Aldrich, St. Louis, MO, USA) at a dose of 1.5 g/kg body weight. Blood glucose levels were measured from tail blood at 0, 15, 30, 60, 90 and 120 min after injection using a glucometer. The area under the curve (AUC) was calculated using the trapezoidal method to evaluate glucose tolerance, insulin sensitivity and pyruvate tolerance.

### 4.5. In Vivo Insulin Stimulation

To assess insulin signaling in adipose tissues, mice were fasted for 4–6 h and intraperitoneally injected with insulin at a dose of 0.5 U/kg body weight. Control mice received an equal volume of saline. Twenty minutes after injection, mice were euthanized, and iWAT, eWAT and BAT were rapidly collected, snap-frozen in liquid nitrogen and stored at −80 °C. Protein samples were prepared for Western blot analysis of insulin signaling molecules, including p-IRβ, total IR, p-AKT and total AKT.

### 4.6. Micro-CT Analysis

Body fat distribution was analyzed using a SkyScan 1176 micro-CT system (Bruker, Kontich, Belgium). Mice were anesthetized and placed in the scanning chamber according to the manufacturer’s instructions. After image acquisition, three-dimensional reconstruction and fat quantification were performed using the manufacturer’s software. Total body fat and abdominal fat content were analyzed and compared between WT and *Irf1* AKO mice.

### 4.7. Metabolic Cage Analysis

Whole-body energy metabolism was assessed using a TSE PhenoMaster metabolic cage system after 16 weeks of HFD feeding. Independent cohorts of HFD-fed WT and *Irf1* AKO mice were used for metabolic cage analysis, β3-adrenergic stimulation, and cold exposure experiments after 16 weeks of HFD feeding. This design was used to avoid potential carryover effects from repeated metabolic challenges. Mice were individually housed in metabolic cages and allowed to acclimate for 24 h before data collection. Oxygen consumption (VO_2_), carbon dioxide production (VCO_2_), energy expenditure (EE), heat production, respiratory exchange ratio (RER), locomotor activity, food intake, and water intake were continuously monitored for 24 h under a 12 h light/dark cycle. Data were analyzed separately according to light and dark phases where appropriate. VO_2_, VCO_2_, EE, and heat production were normalized to lean mass for each individual mouse before statistical analysis.

### 4.8. β3-Adrenergic Stimulation

To evaluate adipose tissue lipolytic response, HFD-fed mice were fasted for 4 h and intraperitoneally injected with the β3-adrenergic receptor agonist CL316,243 (Selleck Chemicals, Houston, TX, USA) at a dose of 1 mg/kg body weight. Blood samples were collected from the tail vein at 0, 5, 15 and 30 min after injection. Plasma was obtained by centrifugation and used for glycerol measurement using a glycerol assay kit (Nanjing Jiancheng Bioengineering Institute, Nanjing, China) according to the manufacturer’s instructions. The glycerol response was evaluated by time-course analysis and AUC calculation.

### 4.9. Cold Exposure Test

To assess adaptive thermogenic capacity, mice were individually placed in a 4 °C environment. Core body temperature was measured using a rectal probe at 0, 1, 2, 3 and 4 h after cold exposure. Mice were monitored throughout the experiment, and body temperature changes were recorded to evaluate cold tolerance.

### 4.10. Western Blot Analysis

Adipose tissues or 3T3-L1 adipocytes were lysed in RIPA buffer containing protease and phosphatase inhibitors. Tissue samples were homogenized on ice and centrifuged at 4 °C to obtain protein lysates. Protein concentration was determined using a BCA Protein Assay Kit (Beyotime Biotechnology, Shanghai, China). Equal amounts of protein were separated by SDS-PAGE and transferred to PVDF membranes. Membranes were blocked with 5% non-fat milk or BSA and incubated with primary antibodies overnight at 4 °C, followed by HRP-conjugated secondary antibodies for 1–2 h at room temperature. Protein bands were detected using ECL reagent (Abclonal Biotechnology, Wuhan, China) and quantified with ImageJ (version 1.54, National Institutes of Health, Bethesda, MD, USA). Target proteins were normalized to GAPDH or α-tubulin. Primary antibodies included IRF1, p-IRβ, IR, p-AKT, AKT, PPARα, PPARγ, SREBF1, PGC-1α, TOMM20, ATP5F1A, and mitochondrial proteins. Detailed antibody information is provided in Supplementary Table S1.

### 4.11. qRT-PCR Analysis

Total RNA was extracted from adipose tissues and 3T3-L1 adipocytes using AG RNAex Pro RNA Extraction Reagent (Aidlab Biotechnology, Changsha, China) according to the manufacturer’s instructions. Frozen tissue samples were homogenized on ice, while cultured cells were directly lysed in RNA extraction reagent after washing with cold PBS. RNA concentration and purity were assessed using a NanoDrop spectrophotometer. Complementary DNA (cDNA) was synthesized using ABScript Neo RT Master Mix (ABclonal Technology, Wuhan, China) following the manufacturer’s protocol. Quantitative real-time PCR (qPCR) was performed using 2 × SYBR Green Pro Taq HS Premix (Aidlab Biotechnology, Changsha, China) on a Roche real-time PCR system. The amplification program consisted of an initial denaturation at 95 °C for 30 s, followed by 40 cycles of 95 °C for 5 s and 60 °C for 30 s, with melt curve analysis to confirm specificity. Relative gene expression was calculated using the 2^−ΔΔCt^ method and normalized to β-actin. All reactions were performed in technical duplicates. Primer sequences are listed in Supplementary Table S2.

### 4.12. mtDNA Copy Number Measurement

Total DNA was extracted from BAT and iWAT using a phenol-chloroform extraction method. Briefly, adipose tissue samples were lysed in DNA lysis buffer containing proteinase K at 55 °C until completely digested. After phenol-chloroform extraction and isopropanol precipitation, DNA pellets were washed, dried and dissolved in TE buffer. DNA concentration and purity were measured using a NanoDrop spectrophotometer. Mitochondrial DNA copy number was quantified by qPCR using mitochondrial gene-specific primers and normalized to nuclear 18S rDNA. Relative mtDNA content was calculated using the 2^−ΔCt^ method.

### 4.13. ATP Measurement

ATP content in BAT, iWAT and 3T3-L1 adipocytes was measured using a luciferase-based ATP assay kit (Abclonal Biotechnology, Wuhan, China) according to the manufacturer’s instructions. Tissue samples were homogenized in ATP lysis buffer on ice and centrifuged at 4 °C. Cell samples were lysed directly after washing with cold PBS. The supernatant was collected for ATP measurement. Luminescence was detected using a microplate reader or luminometer. ATP levels were normalized to protein concentration determined by BCA assay and expressed as relative ATP content.

### 4.14. Oil Red O Staining

Mature 3T3-L1 adipocytes were washed twice with PBS and fixed with 4% paraformaldehyde for 25 min at room temperature. After washing, cells were incubated with 60% isopropanol for 5 min and then stained with freshly prepared Oil Red O working solution (Sigma-Aldrich, USA) for 30–40 min. Excess dye was removed by washing with PBS, and images were captured under a light microscope. For quantification, Oil Red O dye was extracted with isopropanol, and absorbance was measured at 510 nm. Lipid accumulation was expressed relative to the control group.

### 4.15. Glucose Uptake Assay

Glucose uptake in mature 3T3-L1 adipocytes was measured using a Glucose Uptake-Glo assay kit (Promega, Madison, WI, USA) according to the manufacturer’s instructions. Briefly, differentiated adipocytes were serum-starved overnight and then incubated in glucose-free DMEM with or without insulin stimulation. After insulin treatment, cells were incubated with 2-deoxyglucose. The reaction was stopped according to the kit protocol, and luminescence was measured using a microplate luminometer. Glucose uptake was expressed as relative luminescence intensity and normalized to the control group.

### 4.16. Statistical Analysis

Data are presented as mean ± SEM. Statistical analyses were performed using GraphPad Prism software(version 10, GraphPad Software, Boston, MA, USA). Normality was assessed using the Shapiro–Wilk test. Student’s *t*-test was used for comparisons between two groups, and one-way ANOVA followed by appropriate post hoc tests was used for comparisons among multiple groups. For time-course experiments, including glucose tolerance test (GTT), insulin tolerance test (ITT), pyruvate tolerance test (PTT), β3-adrenergic stimulation, and cold exposure experiments, data were analyzed using two-way repeated-measures ANOVA with genotype and time as factors, followed by appropriate multiple-comparison corrections. Area under the curve (AUC) values derived from these experiments were analyzed using Student’s *t*-test or one-way ANOVA as appropriate. Sample sizes varied among experiments because independent cohorts or available biological samples were used for different metabolic, biochemical, and molecular analyses. The exact sample size for each experiment is provided in the corresponding figure legend. A *p* value < 0.05 was considered statistically significant.

## 5. Conclusions

This study suggests that adipocyte IRF1 is associated with insulin signaling, lipid metabolic remodeling, and mitochondrial homeostasis in adipose tissue. Under high-fat diet conditions, adipocyte-specific *Irf1* deficiency was associated with improved systemic glucose tolerance and enhanced insulin-stimulated AKT phosphorylation in white adipose tissues. However, these changes were accompanied by reduced energy expenditure, attenuated β3-adrenergic-induced lipolytic response, altered cold-exposure response, and changes in mitochondrial homeostasis-related markers, including decreased ATP levels, reduced mtDNA content, and downregulation of mitochondrial oxidative phosphorylation-related genes. Overall, these findings highlight a potential dissociation between improved insulin responsiveness and reduced energy expenditure in adipocyte-specific *Irf1* deficiency, suggesting that adipocyte IRF1 may represent a metabolic node linking insulin signaling, lipid metabolism, and mitochondrial homeostasis in obesity-related metabolic disorders.

## Data Availability

The data presented in this study are available from the corresponding author upon reasonable request.

## Supplementary Materials

The following supporting information can be downloaded at: https://www.mdpi.com/article/10.3390/ijms27167332/s1.

## Abbreviations

The following abbreviations are used in this manuscript:

IRF1	Interferon regulatory factor 1
AKO	Adipocyte-specific knockout
WT	Wild type
RCD	Regular chow diet
HFD	High-fat diet
eWAT	Epididymal white adipose tissue
iWAT	Inguinal white adipose tissue
BAT	Brown adipose tissue
GTT	Glucose tolerance test
ITT	Insulin tolerance test
TG	Triglyceride
NEFA	Non-esterified fatty acid
UCP1	Uncoupling protein 1
TOMM20	Translocase of outer mitochondrial membrane 20
