# Supplementary material for: IRF1 in Adipocytes Is Associated with Insulin Signaling and Mitochondrial Homeostasis in Diet-Induced Obesity
Source: Int J Mol Sci. 2026 Aug 17;27(16):7332. doi: 10.3390/ijms27167332 (PMC13513072; doi:10.3390/ijms27167332)
Supplement: Supplementary file 1 [file ijms-27-07332-s001.zip › supplementary.docx.pdf]

S1

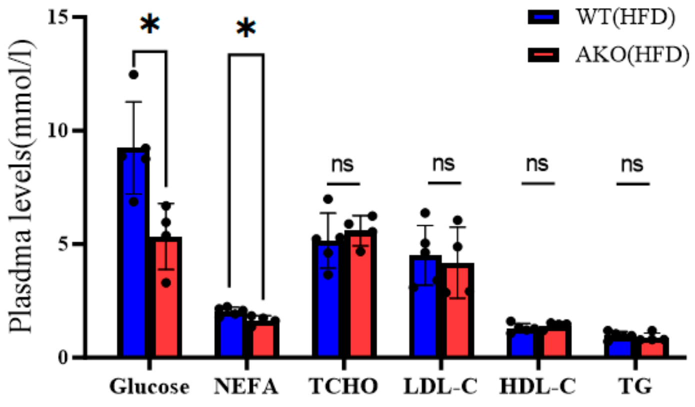

Figure S1. Plasma metabolic parameters in HFD-fed WT and *Irf1* AKO mice. Plasma levels of glucose, non-esterified fatty acids (NEFA), total cholesterol (TCHO), low-density lipoprotein cholesterol (LDL-C), high-density lipoprotein cholesterol (HDL-C), and triglycerides (TG) were measured in HFD-fed WT and *Irf1* AKO mice after overnight fasting (12 h). Data are presented as mean ± SEM. ns, not significant; * $p < 0.05$; $n = 4–5$.

S2

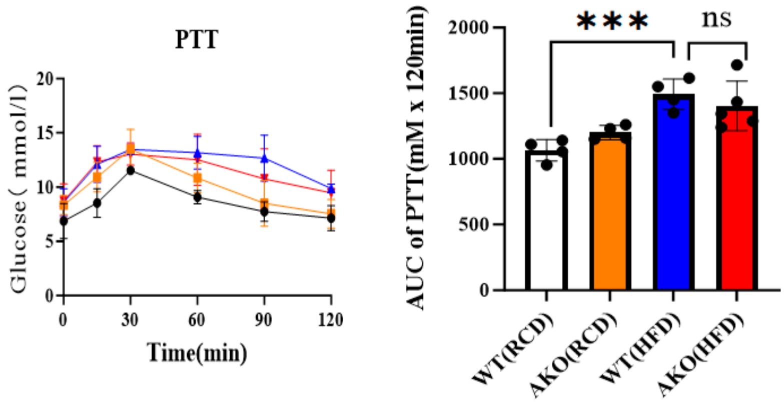

Figure S2. Pyruvate tolerance test (PTT) in WT and *Irf1* AKO mice under RCD and HFD conditions. Left panel shows the time-course of blood glucose levels following pyruvate administration in WT(HFD), AKO(HFD), WT(RCD), and AKO(RCD) mice after overnight fasting (12–14 h). Right panel shows the area under the curve (AUC) of the PTT. Data are presented as mean ± SEM. ns, not significant; *** $p < 0.001$; $n = 4–5$.

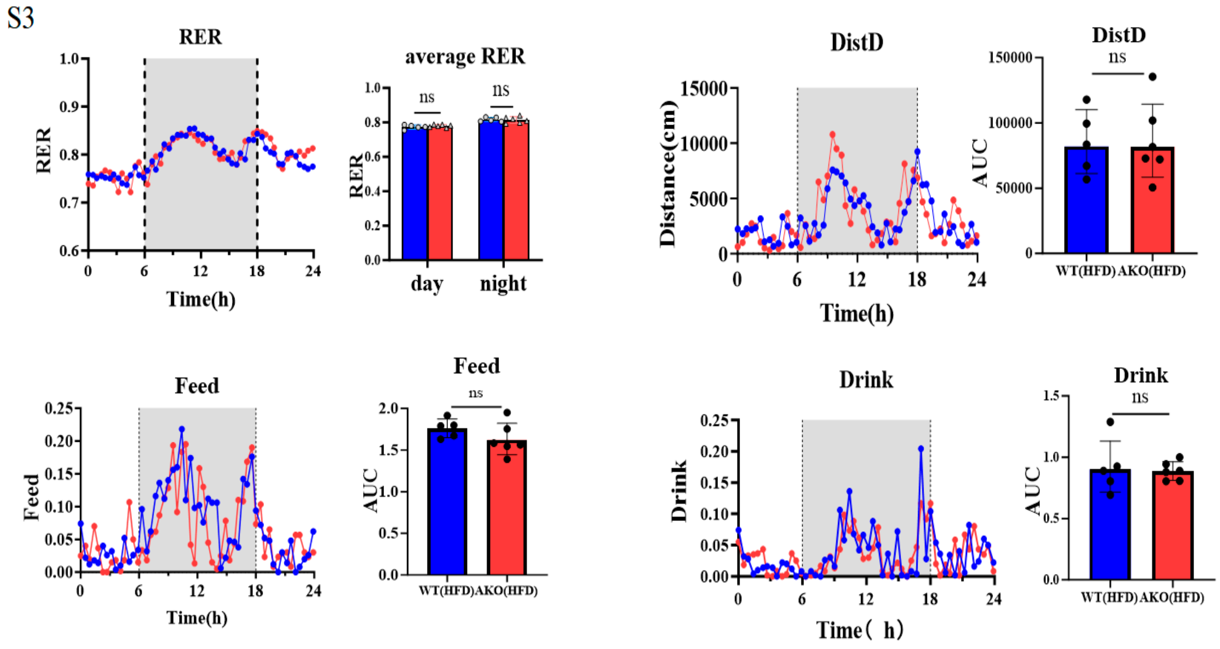

Figure S3. Metabolic cage analysis of respiratory exchange ratio, locomotor activity, food intake, and water intake in HFD-fed WT and *Irf1* AKO mice. Indirect calorimetry was used to assess respiratory exchange ratio (RER), locomotor activity (distance traveled), food intake, and water intake in HFD-fed WT and *Irf1* AKO mice. Left panels show 24 h time-course changes, and right panels show corresponding AUC quantification. Day and night cycles are indicated by shaded areas. Energy expenditure-related parameters were analyzed after normalization to lean mass as described in the Methods section. Data are presented as mean ± SEM. ns, not significant; *n* = 4–6.

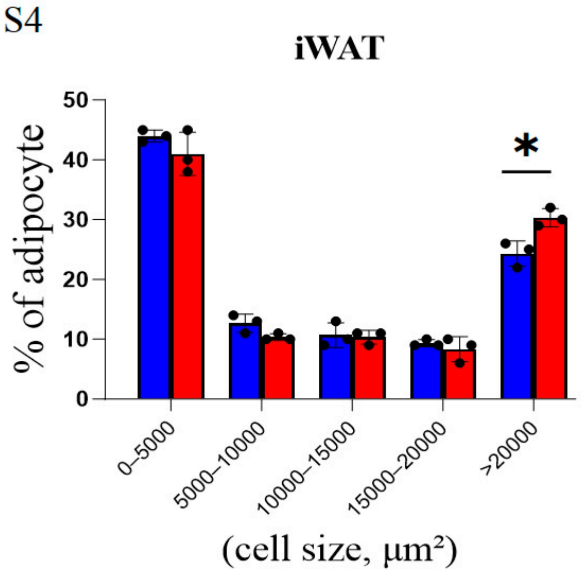

Figure S4. Quantitative analysis of adipocyte area in HFD-fed WT and Irf1 AKO mice. Adipocyte area was quantified from representative H&E-stained sections of iWAT, eWAT, and BAT using ImageJ software (version 1.54, National Institutes of Health, Bethesda, MD, USA). Data are presented as mean ± SEM. ns, not significant; *n* = 3.

S5

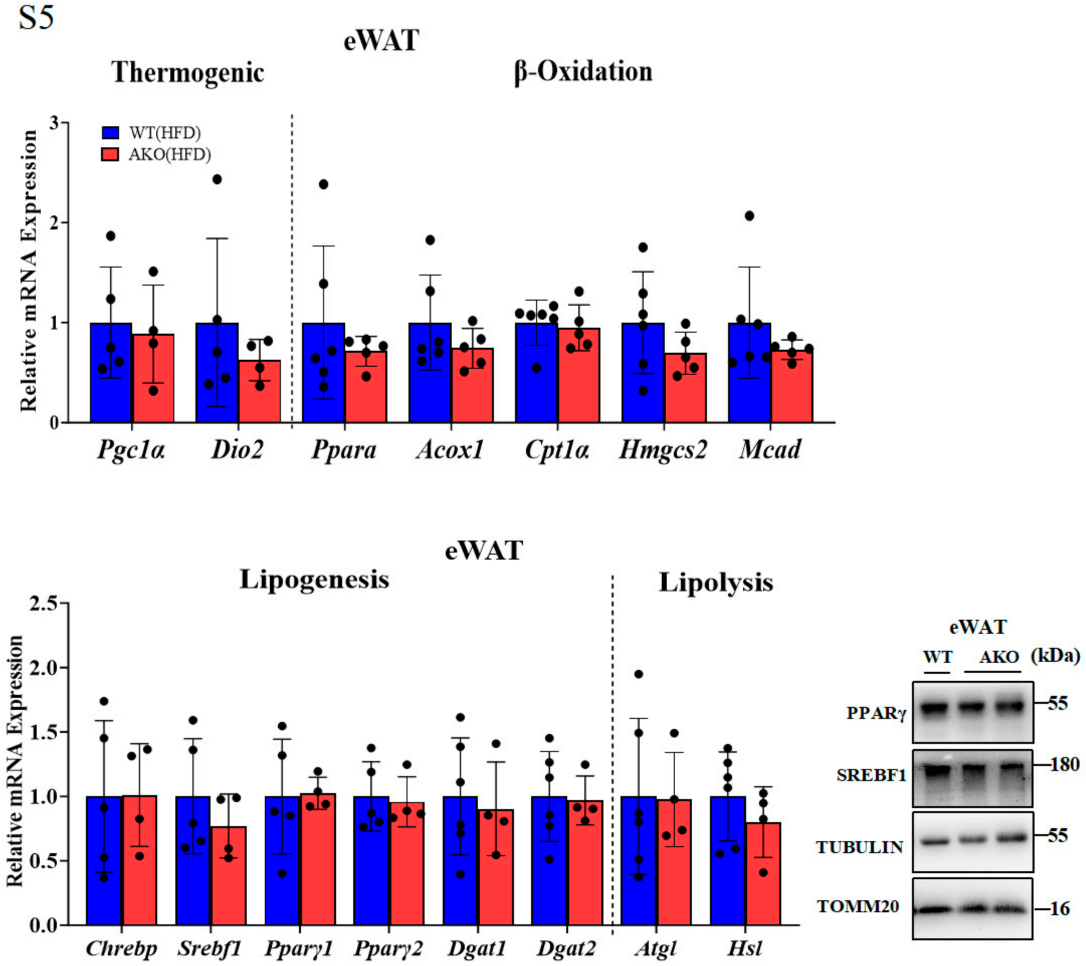

Figure S5. Lipid metabolic markers and TOMM20 protein expression in eWAT under HFD conditions. mRNA expression of thermogenic-, β-oxidation-, lipogenesis-, and lipolysis-related genes was analyzed in eWAT from HFD-fed WT and *Irf1* AKO mice. Representative western blot images of PPARγ, SREBF1, TOMM20, and TUBULIN in eWAT are also shown. Data are presented as mean ± SEM. ns, not significant; *n* = 4–6.

Table S1. Antibodies and their sources

Protein / Antibody	Manufacturer
IRF- 1 (#8478)	Cell Signaling Technology, Danvers, MA, USA
GAPDH (#2118)	Cell Signaling Technology, Danvers, MA, USA
FLAG tag antibody	Proteintech Group, Inc., Rosemont, IL, USA
p-AKTs473 antibody	Cell Signaling Technology, Danvers, MA, USA
AKT antibody	Cell Signaling Technology, Danvers, MA, USA
p-IRβTyr1146 antibody	Cell Signaling Technology, Danvers, MA, USA
IR antibody	Cell Signaling Technology, Danvers, MA, USA
α- tubulin (#3873)	Cell Signaling Technology, Danvers, MA, USA
TOM20 antibody	Zen-BioScience, Chengdu, China
ATP5A antibody	Zen-BioScience, Chengdu, China
NDUFB8 antibody	Zen-BioScience, Chengdu, China

Table S2. Primer sequences used for qPCR analysis

Gene	Forward (5'→3')	Reverse (5'→3')
ms-*Pgc1α*	AGCCGTGACCACTGACAACGAG	GCTGCATGGTTCTGAGTGCTAAG
ms-*Ndufb8*	TGTTGCCGGGGTCATATCCTA	AGCATCGGGTAGTCGCCATA
ms-*sdhb*	CTGAATAAGTGCGGACCTATGG	AGTATTGCCTCCGTTGATGTTC
ms-*Uqcrc2*	AAAGTTGCCCCGAAGGTTAAA	GAGCATAGTTTTCCAGAGAAGA
ms-*Atp5f1a*	TCTCCATGCCTCTAACACTCG	CCAGGTCAACAGACGTGTCAG
ms-*Tfam*	CAGGAGGCAAAGGATGATTC	CCAAGACTTCATTTCATTGTCG
ms-*TOMM20*	CGACCGCAAAAGACGAAGTGAC	GCTTCAGCATCTTTAAGGTCAGG
ms-*β-actin*	GTGACGTTGACATCCGTAAAGA	GCCGGACTCATCGTACTCC
ms-*pparγ1*	CCAACTTCGGAATCAGCTCT	CAACCATTGGGTCAGCTCTT
ms-*pparγ2*	GCTGTTATGGGTGAAACTCTG	ATAAGGTGGAGATGCAGGTTC
ms-*HSL*	ACGCTACACAAAGGCTGCTT	TCGTTGCGTTTGTAGTGCTC
ms-*ATGL*	TTCACCATCCGCTTGTTGGAG	AGATGGTCACCCAATTTCCTC
ms-*C/EBPα*	CAAGAACAGCAACGAGTACCG	GTCACTGGTCAACTCCAGCAC
ms-*Ucp1*	ATACTGGCAGATGACGTCCC	GTACATGGACATCGCACAGC
ms-*Fabp4*	GTGGGCTTTGCCACAAGGAAAT	GGTGATTTCATCGAATTCCACGC
ms-*Adipoq*	AGCCGCTTATGTGTATCGCTCAG	CCCGGAATGTTGCAGTAGAACT
ms-*fasn*	GGAGGTGGTGATAGCCGGTAT	TGGGTAATCCATAGAGCCCAG
ms-*srebf1*	GATGTGCGAACTGGACACAG	CATAGGGGGCGTCAAACAG
ms-*chrebp*	AGATGGAGAACCGACGTATCA	ACTGAGCGTGCTGACAAGTC
ms-*xbp1s*	AGCAGCAAGTGGTGGATTTG	GAGTTTTCTCCCGTAAAAGCTGA
ms-*acc1*	AATGAACGTGCAATCCGATTTG	ACTCCACATTTGCGTAATTGTTG
ms-*acc2*	CCTTTGGCAACAAGCAAGGTA	AGTCGTACACATAGGTGGTCC
ms-*lipin1*	CATGCTTCGGAAAGTCCTTCA	GGTTATTCTTTGGCGTCAACCT
ms-*dgat1*	GCCTTACTGGTTGAGTCTATCAC	GCACCACAGGTTGACATCC
ms-*dgat2*	GCGCTACTTCCGAGACTACTT	GGGCCTTATGCCAGGAAACT
ms-*pparα*	AACATCGAGTGTCGAATATGTGG	CCGAATAGTTCGCCGAAAGAA
ms-*acox1*	GTCTCCGTCATGAATCCCGA	TGCGATGCCAAATTCCCTCA
ms-*cpt1α*	AGGACCCTGAGGCATCTATT	ATGACCTCCTGGCATTCTCC
ms-*hmgcs2*	GAAGAGAGCGATGCAGGAAAC	GTCCACATATTGGGCTGGAAA
